# A simple and efficient tool for trapping gravid *Anopheles *at breeding sites

**DOI:** 10.1186/1756-3305-4-125

**Published:** 2011-07-02

**Authors:** Caroline Harris, Japhet Kihonda, Dickson Lwetoijera, Stefan Dongus, Gregor Devine, Silas Majambere

**Affiliations:** 1 Liverpool School of Tropical Medicine, Pembroke Place, Liverpool, L3 5QA, UK; 2 Ifakara Health Institute, Kiko Avenue, Mikocheni, P.O. Box 78373, Dar es Salaam, Tanzania

## Abstract

**Background:**

No effective tool currently exists for trapping ovipositing malaria vectors. This creates a gap in our ability to investigate the behavior and ecology of gravid *Anopheles*.

**Findings:**

Here we describe a simple trap that collects ovipositing *Anopheline *and *Culicine *mosquitoes. It consists of an acetate sheet coated in glue that floats on the water surface. Ten breeding sites were selected in rural Tanzania and 10 sticky traps set in each. These caught a total of 74 gravid *Anopheles *(54 *An. arabiensis*, 1 *An. gambiae s.s*. and 16 unamplified) and 1333 gravid *Culicines*, in just two trap nights. This simple sampling tool provides an opportunity to further our understanding of the behavior and ecology of gravid female *Anophelines*. It strongly implies that at least two of the major vectors of malaria in Africa land on the water surface during the oviposition process, and demonstrates that *Anophelines *and *Culicines *often share the same breeding sites.

**Conclusion:**

This simple and efficient trap has clear potential for the study of oviposition site choice and productivity, gravid dispersal, and vector control techniques which use oviposition behavior as a means of disseminating larvicides.

## Background

Understanding the ecology and behavior of mosquitoes is a key factor in controlling the diseases they carry [1]. Although malaria vectors have been closely studied for over a century, little is known about their oviposition behavior in the field. This has largely been due to a historical research emphasis on biting behavior and an absence of simple monitoring tools that can be deployed in the field and that will collect gravid females as they search for breeding sites or lay eggs. Resting and oviposition traps are, however, commonly used for the study of other disease vectors. Reflective aluminium plates coated with glue and placed near breeding sites were successful at trapping newly emerged, male, gravid and non gravid *Simulium *species [2]. Sticky traps made of polythene sheets coated with castor oil have been used to monitor and control phlebotomine sandflies [3,4]. Oviposition traps have also been developed for *Culex *[5] and *Aedes *mosquitoes [6-10]. However, to our knowledge, there is no efficient oviposition trap for gravid *Anopheles*, which include malaria and lymphatic filariasis vectors [11]. Such a tool would help in understanding the physiology of gravid females in the wild, distances travelled for oviposition, the characteristics of productive breeding sites and whether gravid mosquitoes deliberately choose these, and for general mosquito surveillance. The primary focus of this study was to develop a simple and affordable tool for monitoring gravid malaria vectors and other mosquito species that share the same breeding sites.

## Study area

**Figure 1:**
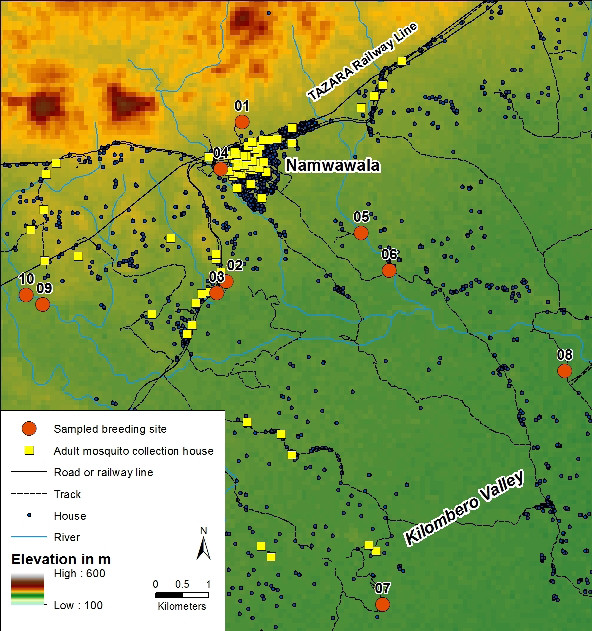
**Map showing the study area and the selected breeding sites**.

This study was carried out in Namwawala village located in the Kilombero valley (8.1^0 ^S and 36.6^0 ^E), south-eastern Tanzania (Figure 1). This is an area of high malaria prevalence and high mosquito density with an estimated 81 infective mosquito bites per person per year [12].

## Experimental design

**Figure 2:**
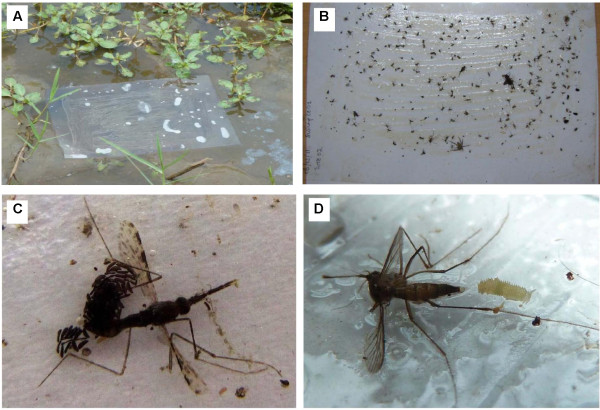
**Oviposition trap**. **A**. Oviposition trap set to float on water, **B**. Mosquitoes and other insects caught on the oviposition trap, **C**. *An. gambiae s.l*. caught on the oviposition trap with her eggs, **D**. *Culicine *caught on the oviposition trap with her egg raft.

The study was conducted in 10 permanent water bodies during the dry season (February 2011). Prior to setting traps, the productivity of the sites was established by conducting 10 larval dips per site using a standard 350 ml dipper (Clarke Mosquito Control Products, Roselle, IL, USA). The trap design consisted of an A4 acetate sheet coated on one side with rat glue (No Rat ^®^, Kollant s.p.a., Italy). The glue was applied evenly on the acetate directly from the tube in a thin layer, leaving a 2 cm glue free perimeter for ease of handling. Traps were labelled with a permanent marker showing the date, water body ID, and trap number. The sticky traps were floated, sticky side up, on the water surface and anchored in place with sticks (Figure 2A). Ten sticky traps were placed at two meter intervals along the periphery of each site. This positioning was based on the assumption that most oviposition occurs around the edges of sites where larval abundance and mosquito emergence is highest [13-15]. Traps were set at 6 pm and collected the following morning at 6 am when they were taken to the laboratory for mosquito identification. In order to confirm that mosquito oviposition had been occurring in the selected water bodies the night the traps were set, larval surveys were performed two days later. Up to thirty larval dips were made in each breeding site around the area where traps had been set to determine the presence or absence of early instar *Culicines *and *Anophelines*. Sticky trap and larval sampling was repeated twice at a one week interval for all 10 breeding sites.

In the laboratory, mosquitoes were morphologically identified to genus and sex and gravidity noted under a dissecting microscope. All mosquitoes were then removed from the traps using a paint brush and paint thinner (Standard Grade Thinner, Orchem, Tanzania) and stored in 96% Ethanol. Adult *Anopheles *were subjected to PCR for species identification, for the *An. gambiae *complex [16] and all negatives for *An. funestus *group [17]. *An. gambiae s.l*. and *An. funestus *group were previously found to make up 100% of the *Anopheline *population caught in CDC light trap catches in the area (94% and 6% respectively, [12]).

## Results

**Figure 3:**
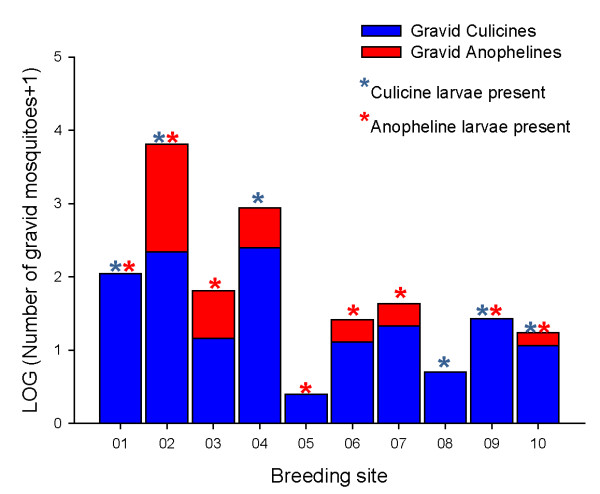
**Average number of gravid mosquitoes caught in two trap nights**. Data for the 10 traps over two trap nights per site have been pooled. Stars indicate the presence of *Anopheles *and *Culicine *larvae two days after oviposition traps were set.

A total of 74 gravid *Anopheles *and 1333 gravid *Culicines *were caught on the sticky traps in two days of sampling, along with seven non-gravid *Anopheles*, one male *Anopheles*, 188 non-gravid *Culicines *and 24 male *Culicines*. Mosquitoes were easily identifiable morphologically using a dissecting microscope, before removal from the trap. The majority of mosquitoes (91% of *Anopheles *and 86% of *Culicines*) caught on traps were gravid, shown by their full and whitish abdomen, with individual eggs visible inside for most species. Occasionally mosquitoes were trapped with eggs laid, producing some rare images of ovipositing field caught mosquitoes (Figure 2B-D). Of the 10 sites sampled, gravid *Anopheles *were caught in six of them at least once in the two trap nights and gravid *Culicines *in all of them (Figure 3). Larval sampling two days after traps were set showed that early stages of *Anopheles *larvae were present in eight of the ten sites and *Culicines *in six of the ten sites (Figure 3), giving an indication of the natural oviposition activity on the sampling night. PCR results show that of the 74 *Anopheline *mosquitoes collected, 54 were *An. arabiensis*, 1 was *An. gambiae s.s*. and 16 did not amplify to either *gambiae *complex or *funestus *group. Morphological Ids on the *Culicines *to sub-genus gave 96% *Culex*, 1.6% *Mansonia*, 0.9% *Aedes *and 1.5% unknown.

## Discussion

This cheap, simple and readily deployable tool will help resolve the long standing challenge of trapping ovipositing mosquitoes, in particular *Anopheles *species. Although this experiment involved only two replicates, it is the result of several months of fine-tuning, which led to the catching of consistently good numbers of *Anophelines *and *Culicines *(Harris & Majambere, unpublished). Oviposition is an important part of mosquito ecology and behavior that remains poorly understood and part of the reason has been the difficulty in sampling gravid mosquitoes [18]. The ability to sample gravid mosquitoes will help researchers understand various aspects of the oviposition process in the field such as the age of ovipositing mosquitoes, the number of eggs produced by wild mosquitoes and the time at which oviposition activity is highest. It could also give insights into the distances gravid mosquitoes travel between a blood source and breeding sites, and whether they deliberately choose those breeding sites that favor development of their offspring. This information could be used in designing vector control measures such as environmental manipulation for mosquito control.

Previous studies on dipterans have shown that reflecting surfaces acting as "collecting mirrors" could be used to trap oviposition seeking black flies [[2] and references therein]. It is possible that oviposition seeking mosquitoes in this experiment were attracted by the reflection from the acetate/glue which could mimic the water surface. The specific point of mosquito oviposition within the breeding site is thought to be non-randomly distributed [13-15]. In our study it is not known whether the trap was more or less attractive than the surrounding water, therefore the trap should be used for comparison between sites rather than estimating oviposition per unit area.

Until now it is not known whether the major malaria vectors *An. gambiae *and *An. funestus *alight on water or hover as they lay their eggs in the field. It is well known that many *Culicines *put their legs into the water during oviposition [19-21], and laboratory studies suggest that *Anophelines *do also [22-24] however no field studies have yet validated this. The success in catching large numbers of *Anopheles *and *Culicine *mosquitoes on the floating sticky traps is a good indication that the species caught here land on or at least touch the water at some point during the oviposition process. When gravid mosquitoes are put under stress (eg. stuck to a sticky trap) it could result in stress induced oviposition [25,26]. Therefore, our photos of mosquitoes stuck to the traps part way through the process of egg laying (Figures 2C and 2D) do not necessarily capture a natural event. However, it does indicate that the legs touch the water surface either during oviposition itself or in preparation for it, as found for *An. atroparvus *[23] and *An. gambiae s.l*. however depending on surface colour [22]. Further investigations using video equipment are expected to bring more clarification on these processes. The suggestion of water contact provided in this paper is an important finding that could be exploited in designing new control measures by manipulating the mosquito-water contact [27,28].

The larval dipping helped to confirm whether mosquitoes were visiting the sites for oviposition the night the traps were set. Due to the difference between trapping methods for gravid mosquitoes and larvae it is difficult to use this data to infer correlation between mosquito densities caught by the two methods.

The Center for Disease Control (CDC) miniature light traps are used for routine monitoring of mosquito densities in the area. Over the two weeks the sticky traps were set a total of 38 CDC light traps were set in surrounding houses collecting a total of 1413 mosquitoes. The subfamily composition of these exactly matches that of the sticky traps, 95% *Culicines*, 5% *Anophelines*, suggesting that sticky traps give an accurate representation of the adult population. Preliminary data in the rainy season show that the trap successfully catches a range of different mosquito species. More rigorous studies are necessary to establish the correlation between species composition from sticky traps and CDC light traps in the rainy season. This study also confirms previous findings observed in East and West Africa that the majority of *Anophelines *and *Culicines *share the same breeding sites, especially during the dry season [13,29].

The current tool was developed in the framework of a project aiming to get mosquitoes to carry insecticides to their breeding sites: the auto-dissemination of insecticides [28]. In order to achieve this, it is important to know which mosquitoes share breeding sites and their oviposition behavior as they select different water bodies to lay eggs in. This trapping tool will help answer these crucial questions in order to design an effective strategy for this novel vector control technique.

## Conclusion

This new sticky trap technique gives a unique opportunity to study the ecology, behavior and physiological state of ovipositing *Anopheles *and *Culicine *mosquitoes. A better understanding of this stage of the mosquito life cycle may result in new opportunities for vector control in manipulating oviposition behaviour.

## Competing interests

The authors declare that they have no competing interests.

## Authors' contributions

GD and SM conceived the study, CH and SM participated in its design and coordination and helped to draft the manuscript. DL, JK, CH and SM participated in data collection. SD mapped the study area. All authors read and approved the final manuscript.
